# Bioerosion of Inorganic Hard Substrates in the Ordovician of Estonia (Baltica)

**DOI:** 10.1371/journal.pone.0134279

**Published:** 2015-07-28

**Authors:** Olev Vinn, Mark A. Wilson, Ursula Toom

**Affiliations:** 1 University of Tartu, Department of Geology, Tartu, Estonia; 2 The College of Wooster, Department of Geology, Wooster, United States of America; 3 Tallinn University of Technology, Institute of Geology, Tallinn, Estonia; Institute of Botany, CHINA

## Abstract

The earliest bioeroded inorganic hard substrates in the Ordovician of Estonia appear in the Dapingian. Hardgrounds are also known from the Sandbian and Katian. Most of the bioerosion of inorganic hard substrates occurs as the boring *Trypanites* Mägdefrau, 1932 along with some possible *Gastrochaenolites* borings. North American hardground borings are more diverse than those in Baltica. In contrast to a worldwide trend of increasing boring intensity, the Estonian record seems to show no increase in boring intensities during the Middle and Late Ordovician. Hardgrounds seem to be more common during the temperate climate interval of the Ordovician calcite sea in Estonia (seven hardgrounds during 15 my) than in the part with a tropical climate (four hardgrounds during 12 my). Bioerosion is mostly associated with carbonate hardgrounds, but cobbles and pebbles broken from the hardgrounds are also often penetrated by *Trypanites* borings. The general diversity of boring ichnotaxa in Baltica increased from one ichnospecies in the Cambrian to seven by the end of Ordovician, showing the effect of the GOBE on bioeroding ichnotaxa. The diversity of inorganic hard substrate borers increased by only two times. This difference can be explained by the wider environmental distribution of organic as compared to inorganic substrates in the Ordovician seas of Baltica, and their more continuous temporal availability, which may have caused increased specialization of several borers. The inorganic substrates may have been bioreroded only by the generalists among boring organisms.

## Introduction

Sedimentary discontinuity surfaces in the Ordovician of Estonia are often marked by mineralization, either with iron minerals or phosphates [1]. All sedimentary discontinuity surfaces are not hardgrounds; many may represent geochemical barriers or were erosional surfaces due to exposure caused by regression. In this study we have counted only hardgrounds that show bioerosion and/or encrustation, which are indications of a true synsedimentarily cemented seafloor surface. Carbonate hardgrounds are common in calcite sea conditions that favored early cementation of carbonate sediments in the seafloor [2]. Hardgrounds form suitable surfaces for bioeroding and encrusting organisms [3]. There was a calcite sea in the Ordovician and hardgrounds were globally abundant during this period [4, 5, 6]. The Ordovician hardgrounds and their bioerosional trace fossils are best known in North America [2]. The western Baltic, especially Sweden, also has a relatively good record of bioerosional trace fossils associated with hardgrounds in the Lower Ordovician [7, 8]. Relatively little is known about the borings in the Estonian and eastern Baltic Ordovician hardgrounds, especially in the Upper Ordovician [9, 10, 11, 12, 13, 14, 15, 16]. Studies of bioerosion of the Ordovician of Estonia have mostly been based on organic substrates such as brachiopods [17, 18] and bryozoans [19, 20].

This paper: 1) records bioeroded inorganic hard substrates in the Ordovician of Estonia, 2) identifies their bioeroding ichnotaxa of inorganic hard substrates, 3) examines trends in the record of this bioerosion, 4) suggests the controls on the bioerosion process of these inorganic hard substrates, 5) finds patterns for the distribution of hardgrounds, and 6) compares the bioerosion in the Ordovician of Baltica with that of North America.

## Previously Studied Hardgrounds

**Fig 1:**
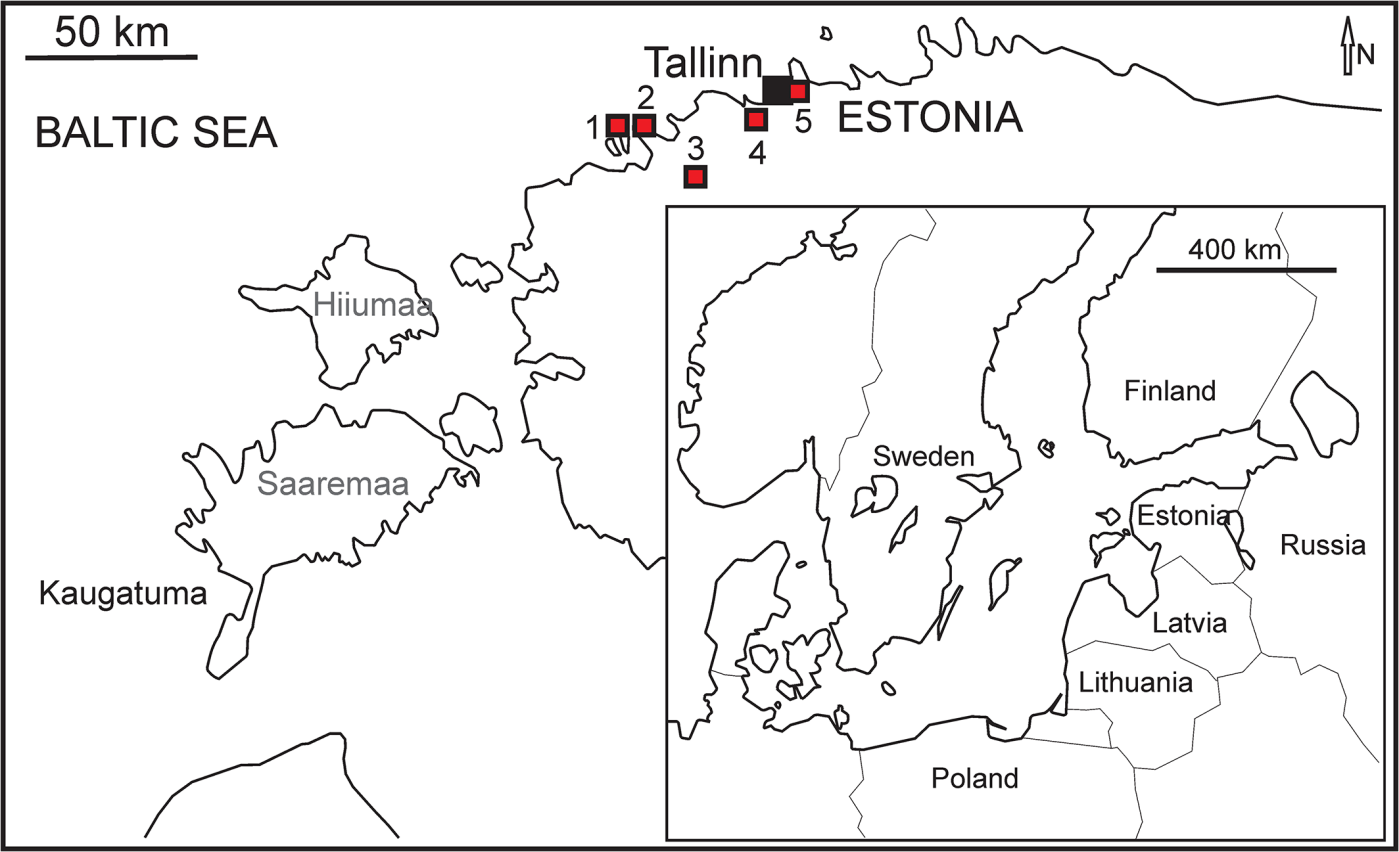
Locality map. Localities in Estonia.1 Väike-Pakri, 2 Cape Pakri, 3 Vasalemma, 4 Alliku, 5 Suhkrumägi.

The Saka hardground forms the base of Volkhov Regional Stage (earliest Dapingian) in NE Estonia (Fig 1); it contains possible *Gastrochaenolites* borings in dolomitized limestone [16]. The density of its bioerosion has not been studied.

**Fig 2:**
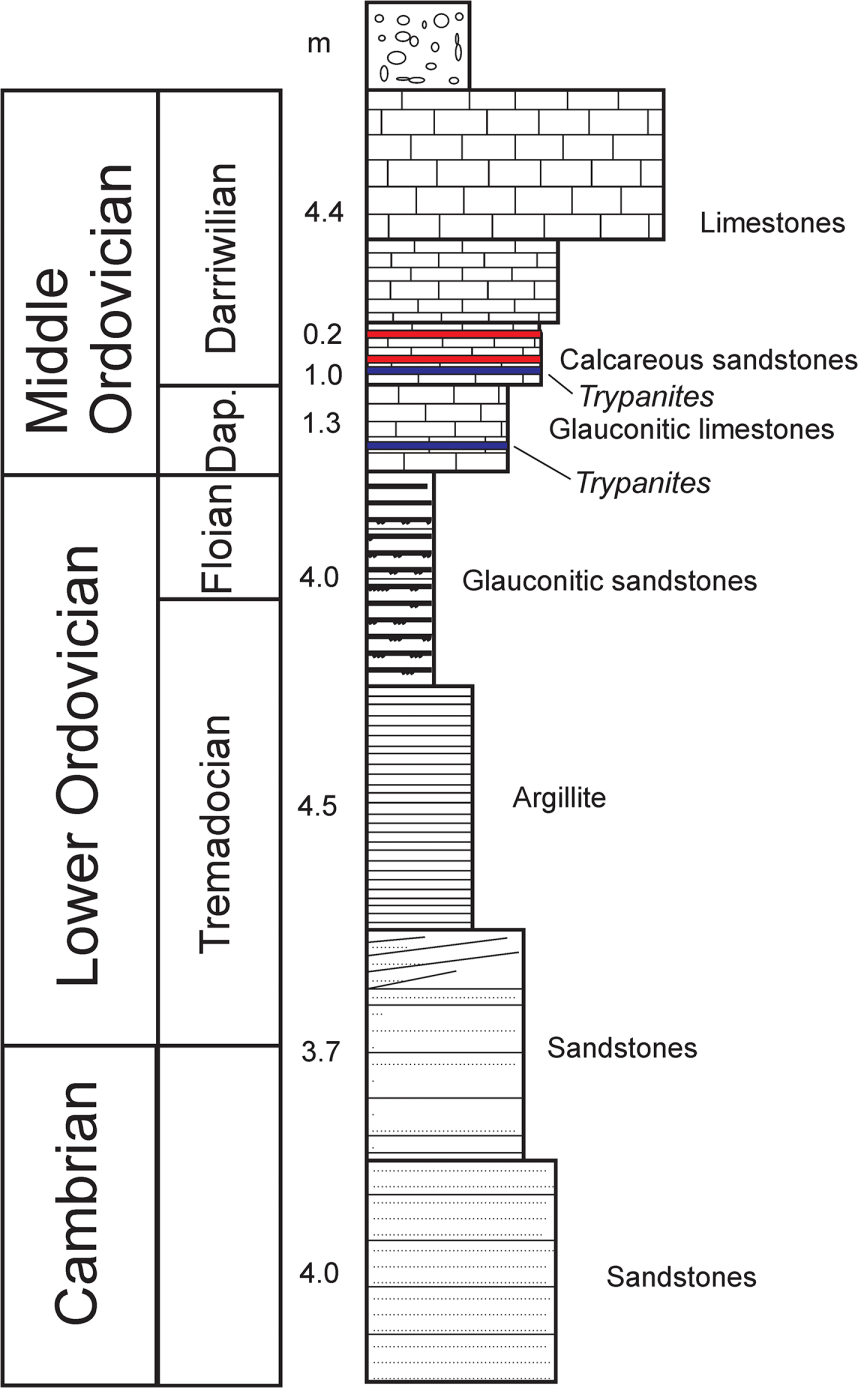
Stratigraphy of Pakri cliff. Modified after Hints et al. (2008). Red-hardgrounds. Blue-cobbles and pebbles.

The Pakri hardground is a surface in the middle of a sandy limestone within the Pakri Formation (Kunda Regional Stage, early Darriwilian) at Pakri Cliff, near Paldiski Town (Figs 1 and 2). This hardground is sparsely encrusted by bryozoans and echinoderms. No bioerosion has been reported [21].

The Väike-Pakri hardground forms the upper surface of a sandy limestone in the Pakri Formation (Kunda Regional Stage, early Darriwilian) on Väike- Pakri Island (Figs 1 and 2). This hardground is sparsely encrusted by large echinoderm holdfasts. Bioerosion has not been recorded [22].

## Geological Background and Localities

During the Ordovician the paleocontinent of Baltica moved from the temperate climatic zone to the subtropical realm [23]. In the Middle and Late Ordovician the climatic change caused an increase in carbonate production and sedimentation rate on the carbonate platform. The appearance of the first carbonate build-ups during the Late Ordovician led to a striking change in the overall character of the Baltic paleobasin in Estonia [24].

The exposure of Ordovician limestones in Estonia forms a wide belt from the Narva River in the northeast to Hiiumaa Island in the west [24]. The total thickness of the Ordovician system in Estonia is moderate, varying from 70 to 180 m [24]. Little bathymetric differentiation characterized the Middle to early Late Ordovician shallow epicontinental sea that covered the western part of the East-European Platform. This basin had an extremely low sedimentation rate [24]. Argillaceous limestones and marls accumulated along the entire extent of the ramp. There was a sedimentological trend of decreasing bioclasts and increasing clay in the offshore direction [23].

Suhkrumägi outcrop (Fig 1): Hardground in the lower part of the Toila Formation (Volkhov Regional Stage, Dapingian); greenish grey dolomitized glauconitic limestones of onshore normal marine origin [1].

Väike-Pakri Island cliff: Hardground (Figs 1 and 2) in the middle of the Toila Formation (Volkhov Regional Stage, Dapingian); greenish grey dolomitized glauconite rich limestones of onshore normal marine origin. Hardground (Figs 1 and 2) in the lower part of the Pakri Formation (Kunda Regional Stage, early Darriwilian); brownish grey calcareous sandstones and sandy limestones of onshore normal marine origin [1].

Alliku quarry: Hardground in the middle of the Kukruse Regional Stage (early Sandbian); intercalation of limestone and oil shale (kukersite) layers of various thicknesses; normal marine origin [1].

**Fig 3:**
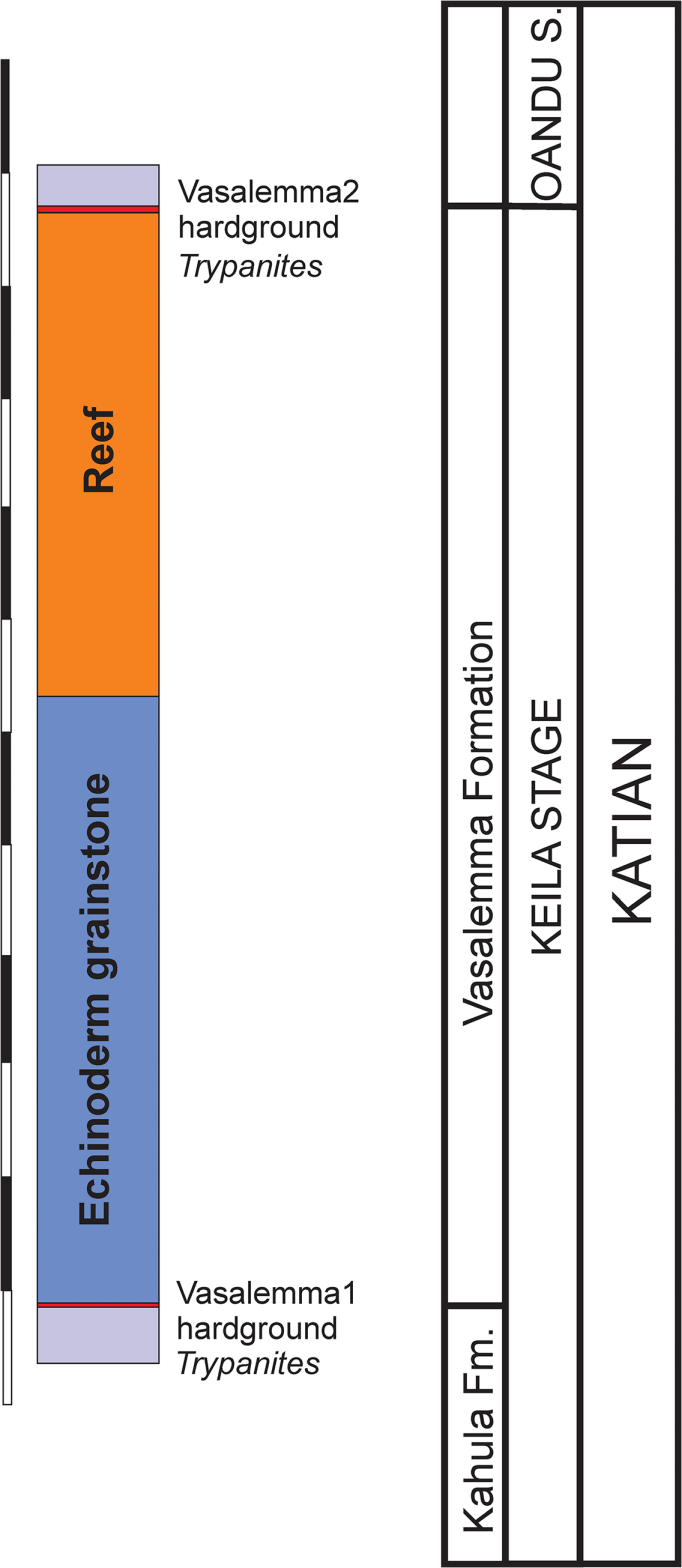
Stratigraphy of Vasalemma quarry. Modified after Kröger et al. (2014).

Vasalemma quarry: Hardground (1) forms the boundary between the Kahula and Vasalemma Formations (Keila Regional Stage, early Katian) (Figs 1 and 3); marly limestones and bioclastic limestones of shallow normal marine origin [1]. Hardground (2) forms the upper boundary of the Vasalemma Formation (Keila Regional Stage, early Katian) (Figs 1 and 3); top of reef bodies, composed of bryozoan framestone-bindstone, echinoderm bindstone, receptaculitid-bryozoan-microbial framestone, and tabulate bafflestone of onshore shallow normal marine origin [1].

Kautla 7034 borehole: Hardground forms the boundary between the Hirmuse Formation and the Tõrremägi Member in the Oandu Regional Stage (early Katian); marly limestones and bioclastic limestones of shallow normal marine origin [1].

Vormsi Island (Saxby costal outcrop): Hardground in the upper part of the Vormsi Regional Stage (late Katian); marly limestones with thin layers of marls of shallow normal marine origin [1].

## Material and Methods

All studied samples were collected on public land where permits are not required, complying with all Estonian regulations. (Collecting hardground samples on public land is not regulated in Estonia.)

### Hardgrounds of this study

**Fig 4:**
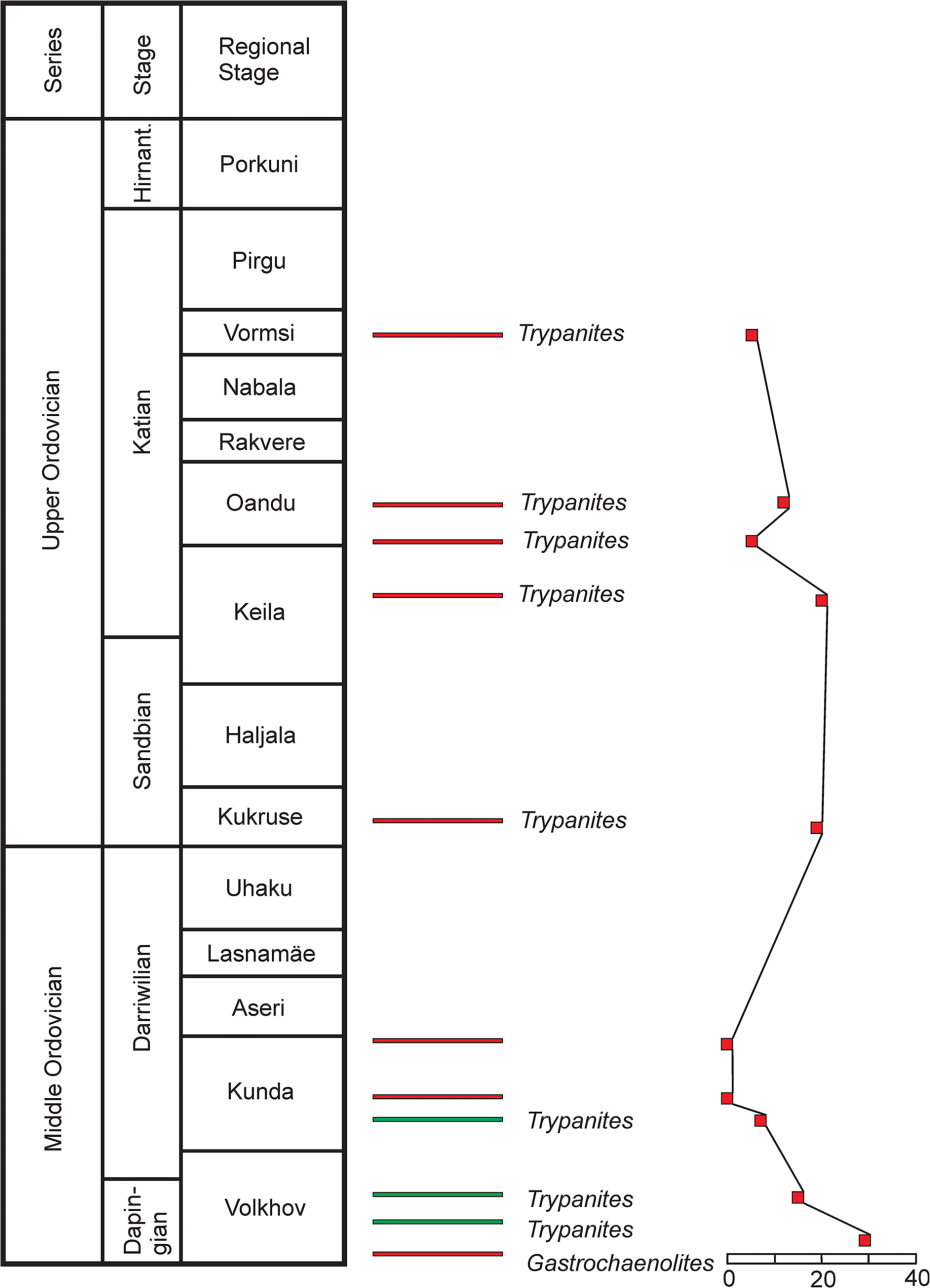
Stratigraphic scheme. The Middle and Upper Ordovician in Estonia with inorganic hard substrates and *Trypanites* shown. Modified after Hints et al. (2008). Scale showing *Trypanites* per 4 cm^2^. One unit on scale = 10 *Trypanites* borings. Red-hardgrounds. Green-pebbles/cobbles.

Two large hardground samples were collected from the Alliku quarry (Kukruse Regional Stage, early Sandbian) (Figs 1 and 4).

Eight large hardground samples were collected from the Vasalemma quarry. This hardground forms the boundary between the Kahula and Vasalemma Formations (Keila Regional Stage, early Katian) (Figs 1 and 4).

Four large hardground samples were collected from the upper surface of reefs in the Vasalemma quarry. This hardground forms the upper boundary of the Vasalemma Formation (Keila Regional Stage, early Katian) (Figs 1 and 4).

Possible hardground or cobble samples from the Kautla 7034 borehole with a total area about 25 cm^2^. This is at the boundary between the Hirmuse Formation and Tõrremägi Member in the Oandu Regional Stage (early Katian) (Fig 4).

Two large hardground samples were collected by Ursula Toom during field work in 2012 on Vormsi Island (Saxby costal outcrop) in the Vormsi Regional Stage (late Katian) (Figs 1 and 4).

### Pebbles and cobbles

Cobbles from the Toila Formation (Volkhov Regional Stage, Dapingian) of Väike-Pakri Island; with diameters 6 to 8 cm. All surfaces are bioeroded.

Pebbles from Suhkrumägi, Toila Formation (Volkhov Regional Stage, Dapingian), with diameters 4 to 6 cm. All surfaces are bioeroded.

Cobbles from the Pakri Formation (Kunda Regional Stage, early Darriwilian) on Väike-Pakri Island; with diameters 7 to 10 cm. All surfaces are bioeroded.

The samples were cleaned and photographed with a scale bar. Borings were measured on calibrated photos. Some samples were longitudinally cut using a rock saw. The sections were thereafter photographed with a scale bar. Borings were counted in 4cm^2^ using a grid drawn on a transparent film, and on calibrated photos. A cm grid was used to measure the area of the studied surface on calibrated photos.

All studied samples are deposited at the Institute of Geology, Tallinn University of Technology, Ehitajate tee 5, Tallinn, Estonia, with specimen numbers (GIT) 381–595, 381–22, 381–1149, 362–114, 362–115, 156–998, 362–98, 362–99, 156–356, 362–95, 362–96, 222–498, 222–449, 222–500, 222–501, 222–502, 222–503, 222–504.

## Results

### Hardgrounds

#### Alliku hardground

**Fig 5:**
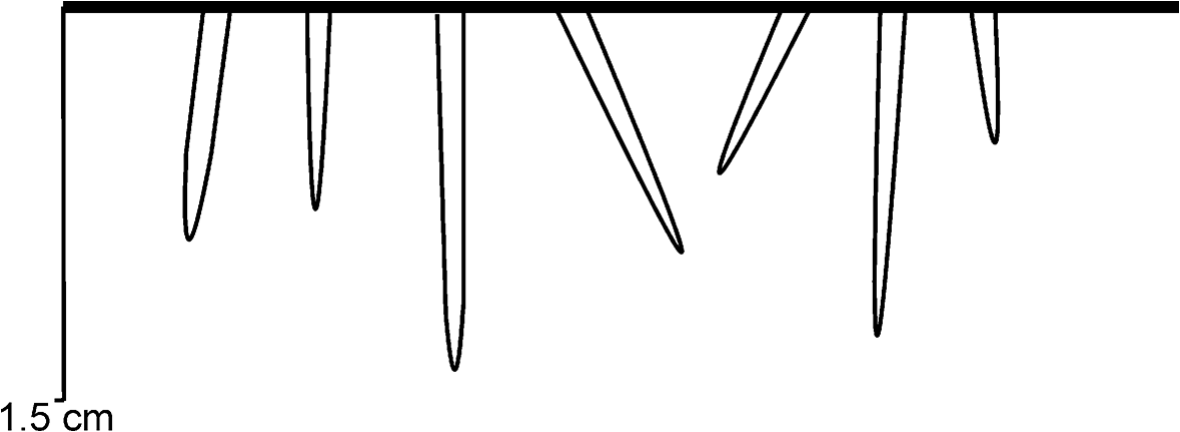
Longitudinal sections of *Trypanites*. A generalization from studied samples.

**Fig 6:**
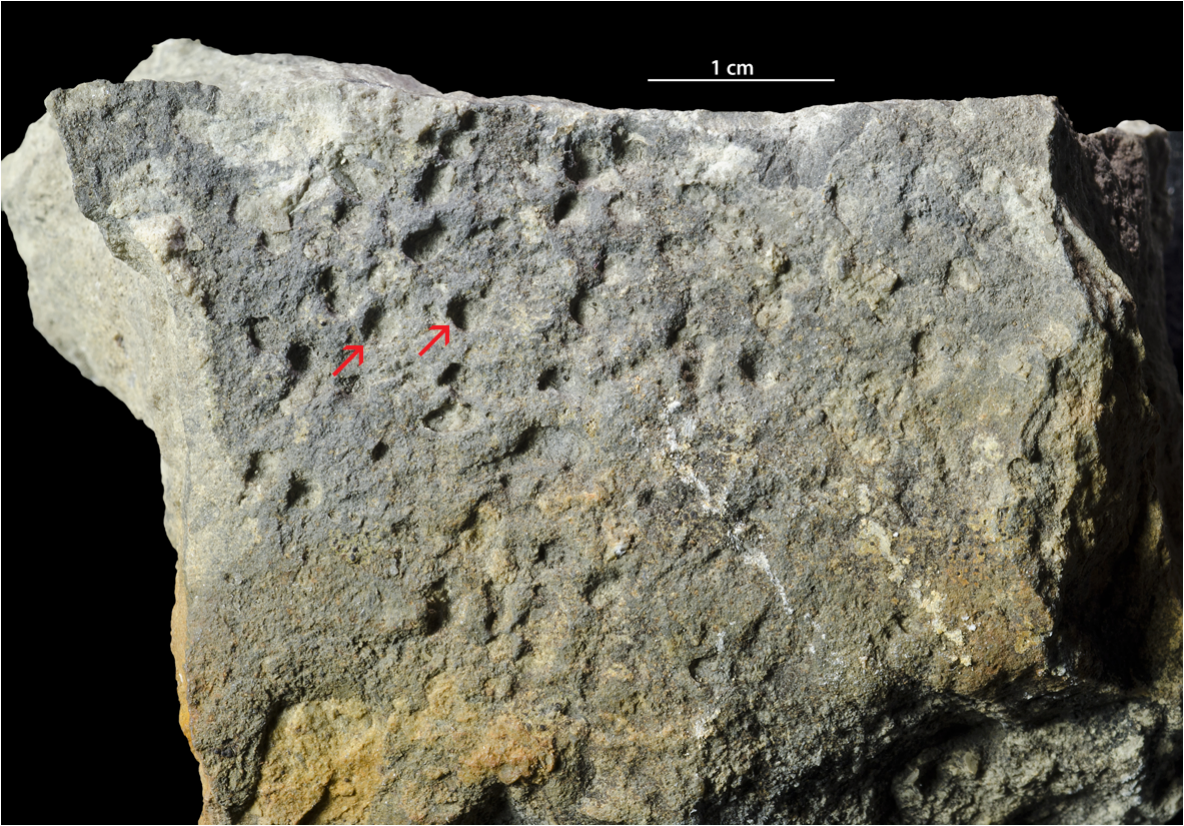
Hardground with *Trypanites*. The Alliku hardground, limestone (early Sandbian) from northern Estonia. GIT 362–115.

**Fig 7:**
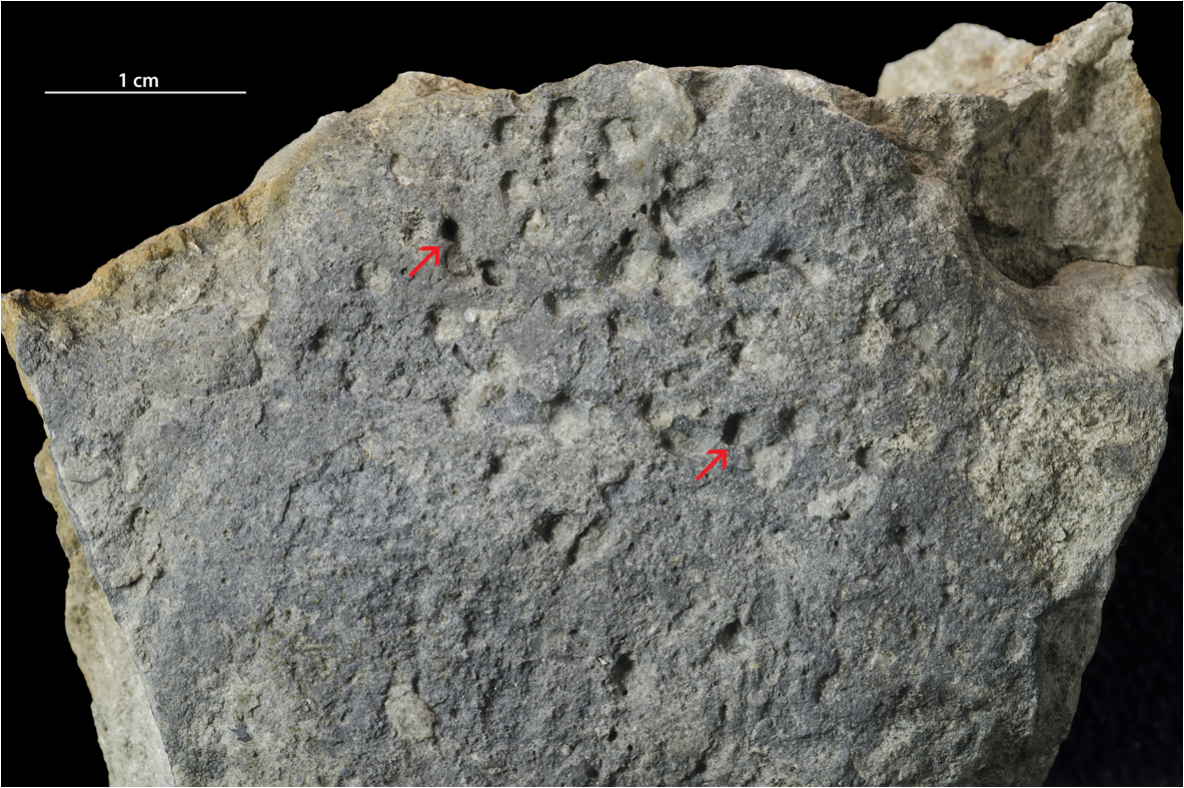
Hardground with *Trypanites*. The Alliku hardground, limestone (early Sandbian) from northern Estonia. GIT 362–114.

The Alliku hardground (early Sandbian) has a relatively even surface with a rough microrelief. The surface of this hardground has a darker grey color than the limestone matrix due to mineralization by iron minerals (presumably pyrite). There are no visible cryptic surfaces. There are no encrusters on the studied hardground slabs. Borings are assigned to *Trypanites* because they are simple cylindrical shafts with single entrances. *Trypanites* borings are very frequent. There are 19 *Trypanites* borings per 4cm^2^. Maximum macroboring density index [25] ranges 3 to 4.*Trypanites* borings have a diameter 0.9 to 2.0 mm (N = 12, mean 1.3 mm, sd = 0.37). The apertures of *Trypanites* are mostly circular, but some can be slightly oval. The distribution of *Trypanites* borings on the hardground surface is patchy (Figs 5, 6 and 7). *Trypanites* borings are not filled with sediment.

#### Vasalemma hardground (lower) 1

**Fig 8:**
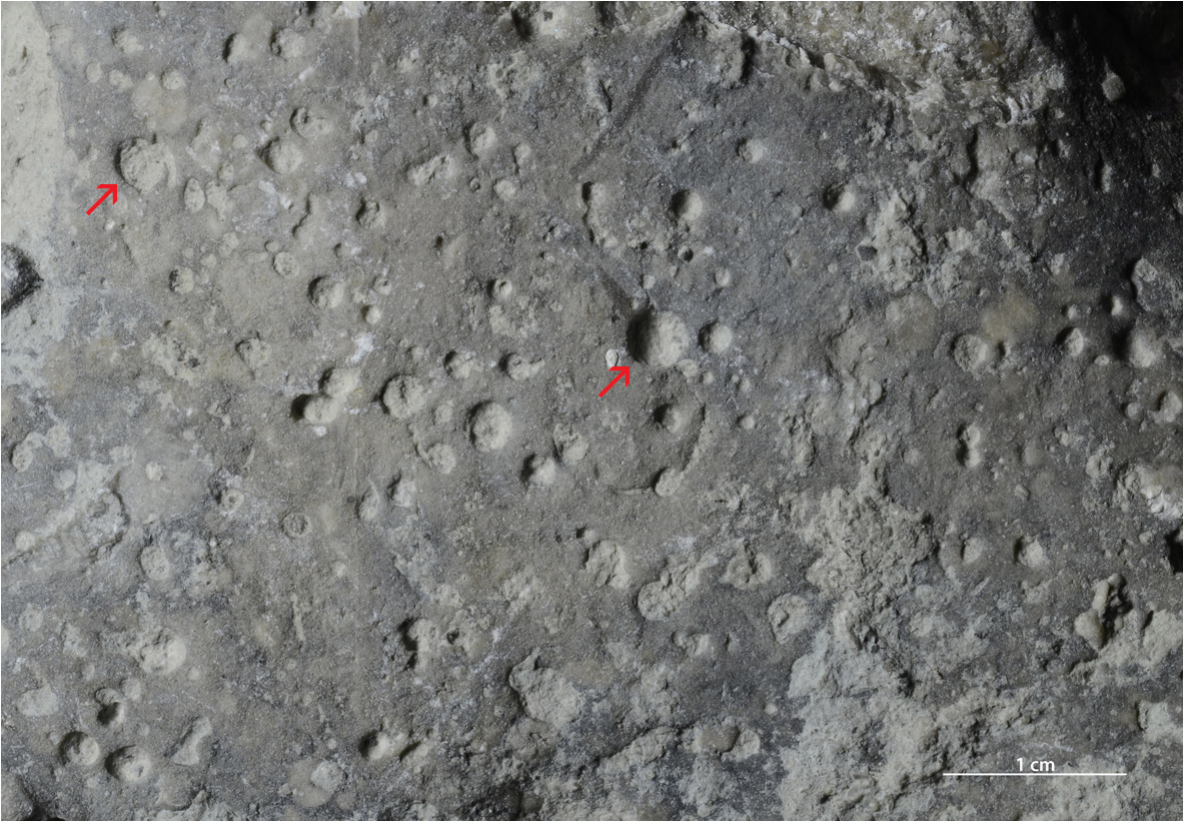
Hardground with *Trypanites*. The Vasalemma hardground 1, micritic-peloidal limestone (earliest Katian) from NW Estonia. GIT 362–95.

**Fig 9:**
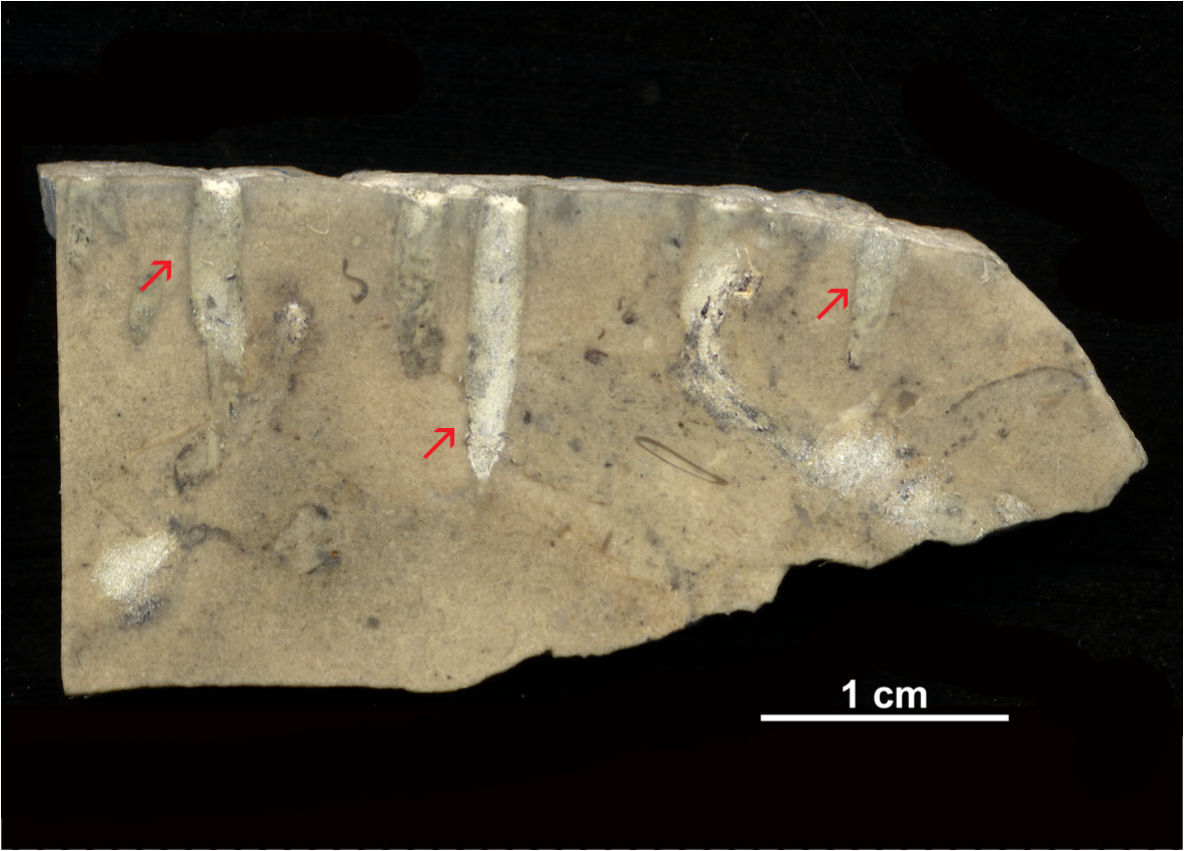
Hardground with *Trypanites*. Longitudinal section through Vasalemma hardground 1, micritic-peloidal limestone (earliest Katian) from NW Estonia. GIT 222–499.

The Vasalemma hardground 1 (earliest Katian) is ripple-marked and has a relatively even surface. The surface of the hardground has slightly darker brownish-grey color than the limestone matrix due to mineralization by iron minerals (presumably pyrite). The surface is eroded and cuts various bioclasts. There are no visible cryptic surfaces. A single eroded cornulitid was found cemented to the hardground. Hints and Miidel [15] reported encrustation by the edrioasteroid echinoderm *Cyathocystis*. *Trypanites* borings are very frequent. The hardground contains 20 *Trypanites* borings per 4cm^2^. Maximum macroboring density index [25] ranges from 3 to 5.The *Trypanites* borings have diameters of 0.5 to 3.3 mm (N = 16, mean 1.5 mm, sd = 0.9). The apertures of *Trypanites* are mostly circular, but some can be slightly oval or subcircular. The distribution of *Trypanites* borings on the hardground is somewhat patchy, but there are no large areas without the borings (Figs 5, 8 and 9). *Trypanites* borings are filled with argillaceous limestone.

#### Vasalemma hardground (above reefs) 2

The Vasalemma hardground 2 (earliest Katian) surface has a darker grey color due to strong pyritization. The rest of rock matrix is light grey. The hardground occurs on the upper surface of a reef limestone layer. The surface is eroded and cuts several bioclasts. Hardground ledges are rounded. Cryptic spaces are formed under the hardground ledges, which are 2 to 9 mm thick. The hardground is sparsely encrusted by cornulitids and bryozoans. *Trypanites* borings are not frequent. *Trypanites* borings range from 0.2 to 2.1 mm in diameter (Fig 5). There are five *Trypanites* per 4 cm^2^ of the hardground upper surface, but some large areas (up to 10 cm^2^) have no borings. The maximum macroboring density index [25] ranges from 0 to 2. The *Trypanites* borings are filled with argillaceous limestone.

#### Possible hardground or cobble from Kautla borehole 7034

The Kautla hardground of early Katian age, the boundary between Hirmuse Formation and Tõrremägi Member in Oandu Regional Stage, is a surface in a limestone with a dark color due to strong pyritization. The rest of rock matrix is light grey in color. *Trypanites* borings are relatively frequent. The hardground surface is relatively smooth and evenly bored by *Trypanites* (Fig 5). There are no encrusters on the studied hardground slab. The hardground contains a maximum 13 *Trypanites* borings per 4cm^2^. The maximum macroboring density index [25] ranges from 2 to 3. *Trypanites* borings have a diameter 0.6 to 4.4 mm (N = 16, mean 2.1 mm, sd = 1.17). The apertures of *Trypanites* are mostly circular, but some can be slightly oval or subcircular. The apertures of some borings are merged. The *Trypanites* borings are filled with argillaceous limestone.

#### Saxby hardground

**Fig 10:**
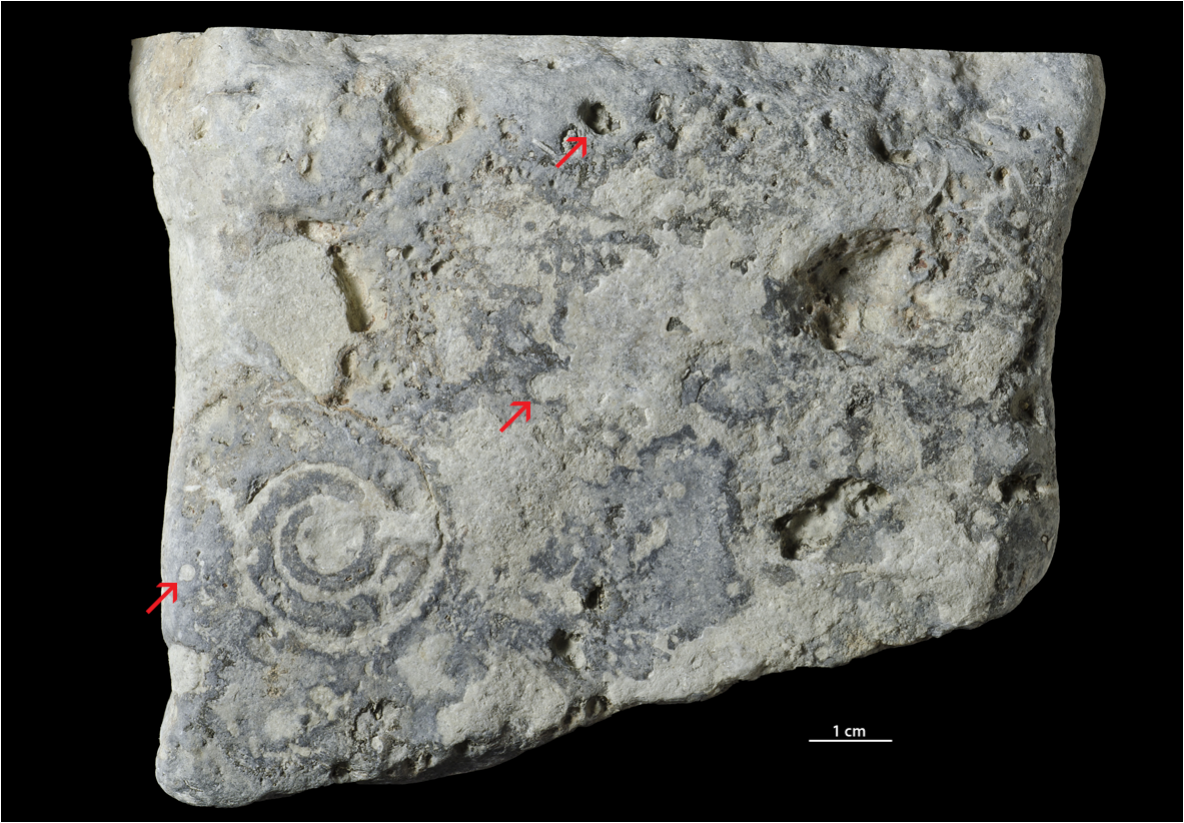
Hardground with *Trypanites*. Saxby hardground (limestone) from the late Katian of Vormsi Island, western Estonia. GIT 362–98.

The Saxby hardground (middle Katian) has an uneven surface and is darker grey than the limestone matrix due to mineralization by iron minerals (presumably pyrite). The surface is eroded and cuts large bioclasts, such as a spiral nautiloid shell. There are no visible cryptic surfaces. There are no encrusters on the studied hardground slabs. *Trypanites* borings are not frequent. It has four *Trypanites* borings per 4 cm^2^. The maximum macroboring density index [25] ranges from 0 to 1. The *Trypanites* borings have diameters 2 to 3.5 mm (N = 7; mean 2.6 mm, sd = 0.62). The apertures of *Trypanites* are circular. *Trypanites* borings are sparsely located on the hardground surface (Figs 5 and 10). The *Trypanites* borings are not filled with sediment.

### Cobbles and pebbles

#### Cobbles from the Toila Formation of Väike-Pakri Island

The studied Dapingian cobble is a platy limestone with slightly lens-shaped profile. Its surface is strongly mineralized (presumably with pyrite) and has a dark color. *Trypanites* borings are very frequent. The surface of the cobble is relatively smooth and evenly bored by *Trypanites* (Fig 5). There is a small holdfast of a possible echinoderm cemented to the cobble. The cobble contains 29 *Trypanites* borings per 4cm^2^. Maximum macroboring density index [25] ranges from 4 to 5. The *Trypanites* borings have diameters 0.6 to 2.8 mm (N = 16, mean 1.7 mm, sd = 0.75). The apertures of *Trypanites* are mostly circular, but some are slightly oval or subcircular and some are merged. The *Trypanites* borings are filled with argillaceous limestone.

#### Pebbles from the Toila Formation of Suhkrumägi

**Fig 11:**
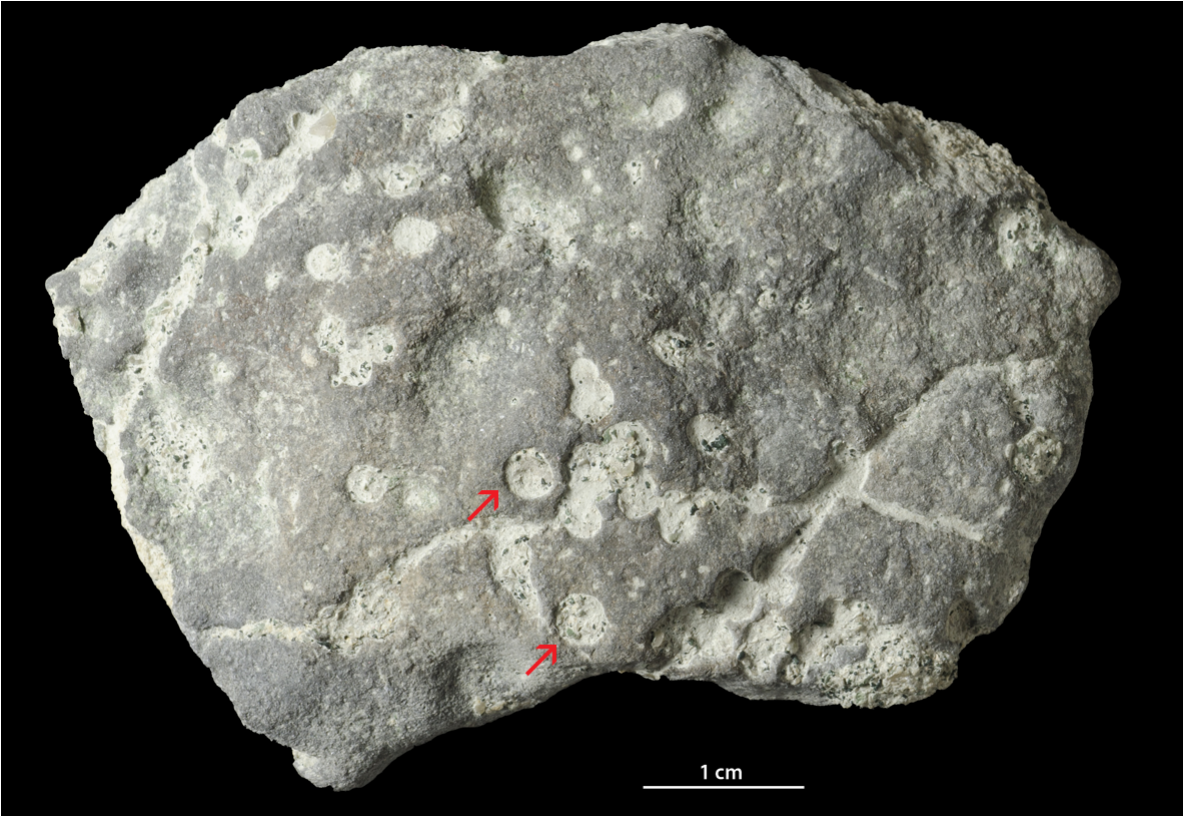
Pebble with *Trypanites*. Toila Formation (Dapingian) from Suhkrumägi, dolomitized glauconitic limestone, northern Estonia. GIT 156–356.

The studied Dapingian limestone pebble has an irregular shape and slightly lens-shaped profile. Its surface is strongly mineralized (presumably with pyrite) and has a dark color. *Trypanites* borings are frequent (Fig 5). The surface of the pebble is slightly bumpy and bored by *Trypanites* from both sides. The distribution of *Trypanites* borings on the pebble is patchy. There are three possible small eroded bryozoans cemented to the pebble. The pebble contains 15 *Trypanites* borings per 4cm^2^. The maximum macroboring density index [25] ranges from 3 to 4. The *Trypanites* borings have diameters 0.7 to 2.9 mm (N = 19, mean 2.3 mm, sd = 0.62). The apertures of *Trypanites* are mostly circular, but some are slightly oval or subcircular, and some are merged (Fig 11). The *Trypanites* borings are filled with argillaceous limestone.

#### Cobbles from Pakri Formation of Väike-Pakri Island

The early Darriwilian limestone cobbles have flat to slightly rounded shape. Their surfaces are mineralized (presumably with pyrite), have a much darker color than their interiors, are slightly bumpy, and are sparsely bored by *Trypanites* on both sides. The surface of one cobble has a relatively rough microrelief. There is a small bryozoan holdfast cemented to one cobble. The cobbles contain maximum seven *Trypanites* borings per 4cm^2^ (Fig 5). Maximum macroboring density index [25] ranges from 1 to 2. *Trypanites* borings have diameters 1.1 to 4.4 mm (N = 12, mean 2.4 mm, sd = 0.87). The apertures of *Trypanites* are mostly circular, but some can be slightly oval or subcircular and a few are merged. The *Trypanites* borings are filled with calcareous sandstone.

### Distribution of bioeroded inorganic hard substrates

Bioeroded limestone pebbles and cobbles occur only in the Middle Ordovician of Estonia, from the early Dapingian (Volkhov Regional Stage) to the early Darriwilian (Kunda Regional Stage) (Fig 4). They are most numerous in the Dapingian (Volkhov Regional Stage). Bioeroded hardgrounds appear in the earliest Dapingian (lower part of the Volkhov Regional Stage) and continue upwards into the middle Katian (Vormsi Regional Stage) (Fig 4). There is a single bioeroded hardground in the Dapingian, two bioeroded hardgrounds in the Darriwilian, a single bioeroded hardground in the Sandbian, and four bioeroded hardgrounds in the Katian.

There are three records of bioeroded hard substrates each in the Dapingian, Darriwilian and early Katian. One more is found in the middle Katian while the Sandbian has just one record.

### Bioerosion of inorganic hard substrates in the Ordovician of Estonia

#### Dimensions

The largest borings occur in the earliest Dapingian (Volkhov Regional Stage) and are possibly *Gastrochaenolites* [16]. On average the largest *Trypanites* borings (mean 2.6 mm) occur in the late Katian (Vormsi Regional Stage) and the smallest (mean 1.3 mm) in the early Sandbian (Kukruse Regional Stage). There is no clear stratigraphic trend in the size distribution of *Trypanites* borings in the Ordovician of Estonia. The size of *Trypanites* borings is not correlated with hard substrate characteristics, such as extent of surface erosion, mineralization or relief. The borings in cobbles, pebbles and hardgrounds have similar sizes.

#### Intensity

The most intensely bioeroded hard substrates occur in the Dapingian (Volkhov Regional Stage) and the least bioeroded in the late Katian (Vormsi Regional Stage). The Dapingian boring intensities are 7–15 *Trypanites* per 4 cm^2^, the Darriwilian boring intensity is 7 *Trypanites* per 4 cm^2^, the Sandbian boring intensity is 19 *Trypanites* per 4cm^2^ and Katian boring intensities are four to 20 *Trypanites* per 4cm^2^. There is no stratigraphic trend in the bioerosion intensity in the Ordovician of Estonia. Both high and low intensities occur in short stratigraphic intervals. Bioerosion intensity does not correlate with the hard substrate characteristics included in this study (see above).

#### Ichnotaxonomy

Most of the bioerosion of Ordovician inorganic hard substrates consists of the boring *Trypanites*. *Gastrochaenolites* is reported only from the earliest Dapingian. It is possible that some of the *Trypanites* reported here may actually belong to *Palaeosabella*, as only a few longitudinal sections of the borings were available.

## Discussion

### Comparison with the other Early Paleozoic inorganic hard substrates

**Fig 12:**
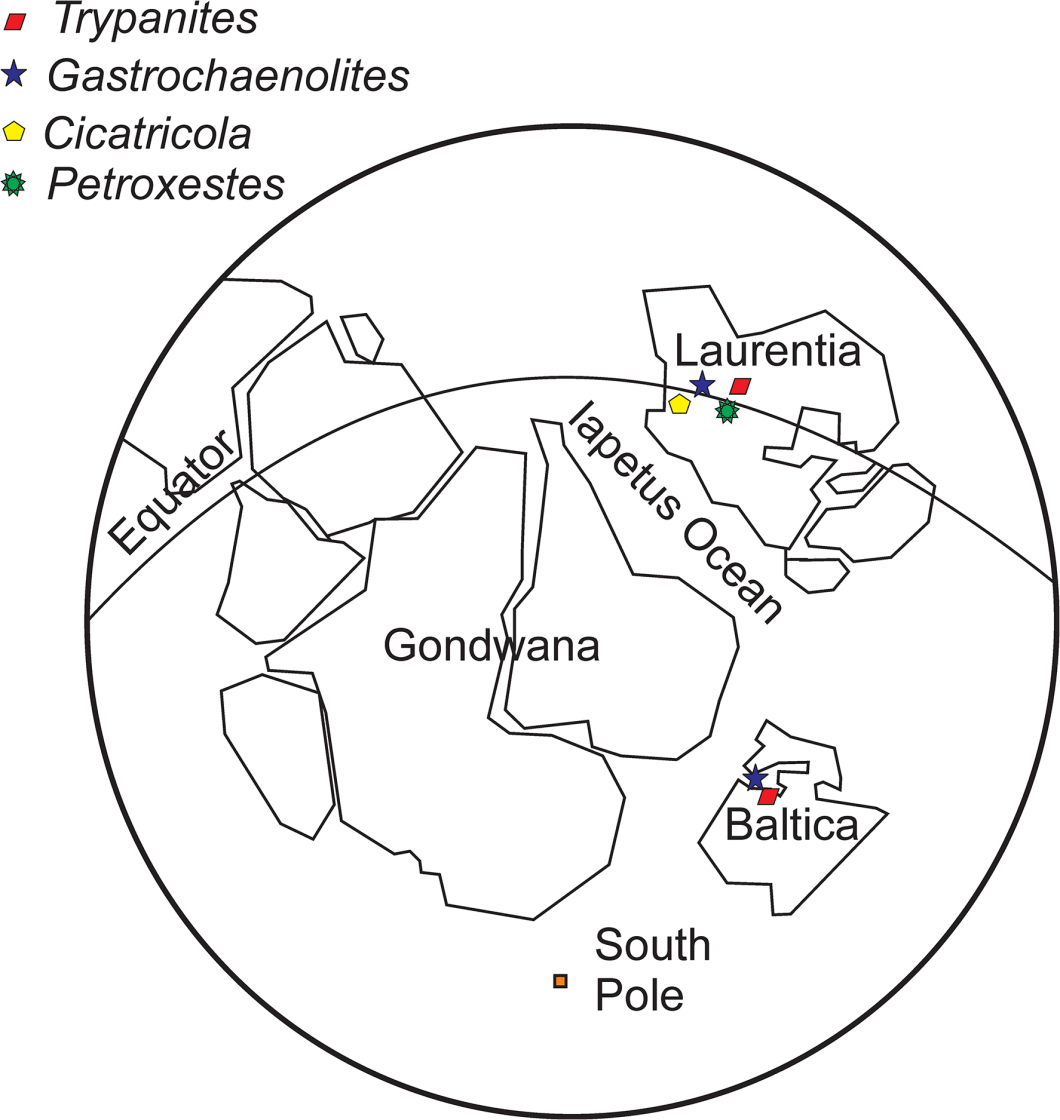
Paleobiogeography of borings. Inorganic hard substrates in the Ordovician. The paleogeographic map of Middle Ordovician is modified after Levin (2006). Data are based on Palmer and Palmer (1977), Wilson and Palmer (1988), Ekdale and Bromley (2001) and present work.

*Trypanites* and possible *Gastrochaenolites* are the only bioerosional trace fossils of inorganic hard substrates common between Estonia (Baltica) and North America (Laurentia) (Fig 12). In addition to these ichnotaxa, North American hardground faunas include *Petroxestes* and *Cicatricula* [26]. *Petroxestes* is a surficial elongate boring produced by bivalves and is known from hardgrounds from the Ordovician of North America [26, 27] (Fig 12). Similarly, *Cicatricula* is found on Middle Ordovician hardground surfaces in Iowa [28] (Fig 12). It is a shallow ramifying boring. It is likely that North American hardground borings were more diverse than those in Baltica. This could be explained by different environmental conditions, such as substrate texture, oxygen levels, nutrient content, depositional energy and sedimentation rate, caused by climatic differences during most of the Ordovician. Most of North America was located in the tropics during the Ordovician while Baltica was located in a temperate climate zone from the earliest Ordovician to the Sandbian. An alternative explanation would be collecting bias, as the North American hardground faunas have been more thoroughly studied than the Estonian ones.

*Trypanites* borings are most intense in the Late Ordovician of North America [27]. However, the Estonian inorganic hard substrates do not show a consistent trend in boring intensities (i.e., number of borings per unit of hard substrate area) during the Middle and Late Ordovician, but this could be explained by the insufficient sample size and different exposure times of Middle Ordovician and Late Ordovician substrates. The sedimentation rates were lower in the Middle Ordovician of Estonia than in the Late Ordovician [1]. Thus, it is likely that the Middle Ordovician hard substrates were in general exposed longer to bioeroding organisms than the Late Ordovician substrates.

The dimensions of Estonian *Trypanites* borings in the inorganic hard substrates do not differ from analogous records from the Ordovician of North America and other regions of Baltica [25].

### Stratigraphic distribution of hardgrounds

Hardgrounds are often broken into cobbles and larger pebbles due to hydrodynamic activity. The above described bioeroded cobbles and pebbles from the Ordovician of Estonia are interpreted here as the reworked remains of contemporaneous hardgrounds because of their plate-like shape. The earliest bioeroded hardground occurs almost immediately after the beginning of carbonate sedimentation in the Paleobaltic Basin (Fig 4). However, the lack of hardgrounds in the latest Ordovician (latest Katian and Hirnantian) of Estonia needs an explanation. This could be due to less favorable conditions for hardground formation or by higher sedimentation rates in the latest Ordovician of Estonia. Alternatively, it could reflect a collecting bias, but this is unlikely as the whole Ordovician of Estonia has been equally well sampled.

The temporal distribution of hardgrounds reveals a possible trend (Fig 4). There are seven hardgrounds in the Dapingian to early Sandbian (15 my) and only four hardgrounds in the late Sandbian to Hirnantian (12 my) (Fig 4). This may indicate that there were unfavorable conditions for hardground formation during most of the Katian and Hirnantian. The uneven stratigraphic distribution of bioeroded inorganic hard substrates is explained by the varying availability of these substrates in the Ordovician of Estonia.

### Bioerosion trends in organic hard substrates

Among Ordovician organic hard substrates in Estonia, bioerosion has been studied only in bryozoans [19, 20] and brachiopods [17, 18] (Table 1). Bioerosion has a relatively good stratigraphic record only for bryozoans in the Ordovician of Estonia, but no trends can be detected. Data published by Wyse Jackson and Key [19] show that both in the Middle Ordovician (N = 25) and the Late Ordovician (N = 115) 80% of bryozoans were bored by *Trypanites*. However, their data for the early Middle Ordovician are insufficient for statistical analysis, and the Late Ordovician record lacks the middle Katian to Hirnantian part of the section, so it is impossible to draw any firm conclusions. The latest Sandbian to earliest Katian (Keila Regional Stage) is characterized by relatively intense bioerosion (90% of bryozoan specimens) [19]; similarly, the Vasalemma 1 hardground has relatively high bioerosion intensity (20 *Trypanites* borings per 4 cm^2^). It is possible that this may indicate slightly more favorable environmental conditions for bioerosion in the latest Sandbian to earliest Katian (Keila) time. Bryozoan data suggest that bioerosion intensities were similar in the Middle and Late Ordovician of Estonia.

**Table 1:** Comparison of *Trypanites* borings in organic and inorganic substrates. The Ordovician of Estonia.

Character	Organic hard substrates		Inorganic hard substrates	
	Bryozoans	Brachiopods	Cobbles/pebbles	Hardgrounds
Diameter (mm)	Mean 2.1–2.6	0.2–1.6, mean 0.2–1.0	0.6–4.4, mean 1.7–2.4	0.2–4.4, mean 1.3–2.6
Length (mm)	Mean 4.9–7.1	2–8	7–12	6–15
Morphology	Simple cylindrical	Simple cylindrical	Simple cylindrical	Simple cylindrical
Typical bioerosion intensity	Medium to high	Low	High	Medium
Bioerosion environment	Shallow marine, carbonate platform (Onshore to offshore)	Shallow marine, carbonate platform (Onshore to offshore)	Shallow marine, carbonate platform (Onshore to offshore)	Shallow marine, carbonate platform (Onshore to offshore)

### Climatic change, bioerosion and hardground formation

A possible explanation for the uneven stratigraphic distribution of hardgrounds in the Ordovician of Estonia may be in sedimentation rate differences. The Dapingian and early Darriwilian are characterized by low net sedimentation in the Paleobaltic basin [1]. The Early Katian Vasalemma Formation may have similarly been affected by low sedimentation rates. The sedimentation pattern during the latest Keila, Sandbian/Katian boundary (Vasalemma Formation) interval has been described as unique as it represents a time of pronounced non-deposition over most parts of northern Estonia [29].

The Ordovician was a time of calcite seas, which produced favorable conditions for hardground formation [30]. According to a recent review by Balthasar and Cusack [31], calcite sea intervals were characterized by the co-precipitation of aragonite and calcite in environments above 20°C. They concluded that continuous prominence of aragonite precipitation in Phanerozoic warm-water environments could explain the Phanerozoic increase of aragonite over calcite skeletal composition in calcifying organisms. Thus, the geochemical regimes in tropical and temperate calcite seas were different. During the Ordovician, Baltica moved from a temperate climate (Tremadocian to Sandbian) to the tropics (late Katian and Hirnantian). Hardgrounds seem to be more common in the temperate climate part of the Ordovician calcite sea in Estonia (seven hardgrounds during 15 my) than in the tropical portion of the record (four hardgrounds during 12 my). If there is a trend, it is possible that the temperate climate geochemical regime of calcite seas could have been more favorable for hardground formation than the tropical equivalent. Future studies should show whether there is a climate-related trend in the distribution of hardgrounds in Baltica.

There seems to be no correlation between hardground formation and minor climatic perturbation, such as the GICE (Guttenberg δ13C excursion), for example, in the Ordovician of Estonia. There is no event associated clearly with numerous hardgrounds.

### GOBE and the Ordovician Bioerosion Revolution

The Great Ordovician Biodiversification Event (GOBE) is reflected in the record of bioerosional trace fossils and is termed the Ordovician Bioerosion Revolution. During the Ordovician Bioerosion Revolution, the diversity of boring ichnotaxa dramatically increased. There are two macroborings in the Cambrian, which contrasts with nine ichnotaxa known from the Ordovician [26, 20].

The evolution of bioerosion in Baltica follows a similar pattern, but there are some differences in diversity and taxonomic composition between North America and Baltica [20, 26]. The following borings occur in the Ordovician of Baltica: *Trypanites*, *Palaeosabella*, *Gastrochaenolites*?, *Osprioneides*, *Ropalonaria*, and *Sanctum* [17, 19, 20, 26]. *Trypanites* and *Gastrochaenolites* are the only known inorganic hard substrate borings in the Ordovician of Baltica. Interestingly, when the general diversity of boring ichnotaxa in Baltica increased from one to seven during the Ordovician, the diversity of inorganic hard substrate borings increased only two times. This difference in diversity increase may be explained by the wider environmental distribution of organic substrates as compared to inorganic ones in the Ordovician seas of Baltica, and their continuous temporal availability. These factors may have encouraged specialization of several borers. The inorganic substrates were probably bored only by the generalists.

## Conclusions

- Bioeroded hardgrounds, limestone pebbles and cobbles are common in the Ordovician of Estonia.
- Bioerosional trace fossils in inorganic hard substrates are less diverse than those on organic skeletons in the Ordovician of Baltica.
- The major bioerosional ichnotaxon of inorganic hard substrates is *Trypanites* in the Ordovician of Baltica.
- Hardground borings in North America are more diverse than those in Estonia (Baltica).
